# Comparison of non-coplanar optimization of static beams and arc trajectories for intensity-modulated treatments of meningioma cases

**DOI:** 10.1007/s13246-021-01061-8

**Published:** 2021-10-07

**Authors:** Tiago Ventura, Humberto Rocha, Brigida da Costa Ferreira, Joana Dias, Maria do Carmo Lopes

**Affiliations:** 1 Physics Department of University of Aveiro, Aveiro, Portugal; 2 Medical Physics Department of the Portuguese Oncology Institute of Coimbra Francisco Gentil, EPE, Coimbra, Portugal; 3 Institute for Systems Engineering and Computers at Coimbra, Coimbra, Portugal; 4 Economy Faculty of University of Coimbra and Centre for Business and Economics Research, Coimbra, Portugal; 5 Instituto de Biofísica e Engenharia Biomédica, Faculdade de Ciências da Universidade de Lisboa, Lisboa, Portugal; 6 I3N Physics Department of University of Aveiro, Aveiro, Portugal

**Keywords:** Beam angle optimization, Non-coplanar, Arc trajectory, VMAT

## Abstract

Two methods for non-coplanar beam direction optimization, one for static beams and another for arc trajectories, were proposed for intracranial tumours. The results of the beam angle optimizations were compared with the beam directions used in the clinical plans. Ten meningioma cases already treated were selected for this retrospective planning study. Algorithms for non-coplanar beam angle optimization (BAO) and arc trajectory optimization (ATO) were used to generate the corresponding plans. A plan quality score, calculated by a graphical method for plan assessment and comparison, was used to guide the beam angle optimization process. For each patient, the clinical plans (CLIN), created with the static beam orientations used for treatment, and coplanar VMAT approximated plans (VMAT) were also generated. To make fair plan comparisons, all plan optimizations were performed in an automated multicriteria calculation engine and the dosimetric plan quality was assessed. BAO and ATO plans presented, on average, moderate global plan score improvements over VMAT and CLIN plans. Nevertheless, while BAO and CLIN plans assured a more efficient OARs sparing, the ATO and VMAT plans presented a higher coverage and conformity of the PTV. Globally, all plans presented high-quality dose distributions. No statistically significant quality differences were found, on average, between BAO, ATO and CLIN plans. However, automated plan solution optimizations (BAO or ATO) may improve plan generation efficiency and standardization. In some individual patients, plan quality improvements were achieved with ATO plans, demonstrating the possible benefits of this automated optimized delivery technique.

**Supplementary Information:**

The online version contains supplementary material available at 10.1007/s13246-021-01061-8.

## Introduction

In radiation therapy, non-uniform intensity field techniques are well-established for almost all cancer pathologies since they allow the delivery of highly conformal dose distributions to the target(s) while minimizing the injury to the organs-at-risk (OAR). The calculation of non-uniform beam intensities is done using inverse planning, where plan objectives are specified by means of physical or biological descriptors in an objective function that guides the fluence map optimization (FMO) process [1]. Usually, the planning optimization is performed through a trial-and-error manual tuning of plan parameters until an acceptable plan is obtained.

For conventional C-arm linear accelerators, this type of treatment techniques can be delivered through multiple modulated static or dynamic radiation fields (intensity-modulated radiation therapy—IMRT) or through continuously modulated radiation arcs combining the variation in dose rate, gantry speed and aperture shape (volumetric modulated arc therapy—VMAT). For most tumour sites, equivalent plan quality can be achieved by IMRT or VMAT. Nevertheless, VMAT treatments are usually more efficient requiring fewer monitor units and thus shorter delivery times [2].

Most of IMRT and VMAT treatments are still performed using equidistant coplanar static beams or coplanar arcs. As these approaches typically obtain acceptable treatment plans, beam angle optimization methods are still not popular among the clinical community. However, when non-coplanar geometries are included in the optimization, improved normal tissue sparing, target conformity and steeper dose gradients can be achieved. Indeed, an appropriate beam assembly or arc trajectory selection may lead to improvements in the dosimetric quality of the plans [3, 4].

Beam angle optimization is complex, time-consuming and it often presents non-intuitive solutions. Mathematically, it is defined as a highly non-convex multi-modal optimization problem with many local minima [5–7], requiring optimization methods that avoid being trapped in a local minimum. For IMRT, the beam angle optimization problem considering non-coplanar geometries has been extensively studied for brain [3, 8–10], head-and-neck [10–12, 14–16], lung [17], gastric [12], liver [14, 18, 19], pancreas [10], cervix [14] and prostate [10, 12, 13] sites. The reported beam angle optimization methods can be grouped into two classes. In the first class, beam angle selection and the FMO processes are independent and are addressed sequentially. The beam angle optimization process is normally driven by geometrical or dosimetric metrics or by methods that require some prior knowledge of the problem [3, 12, 13]. These methods are computationally efficient, but the resultant beam angle ensemble does not guarantee the optimality of the plan solution. In the second class of methods, the beam angle optimization and the FMO processes are simultaneously solved. The FMO is used to guide the beam angle optimization by assessing the goodness of the plan. The beam angle optimization problem can be formulated by considering a combinatorial search for the best ensemble over a discretized space search or by a continuous space search optimization. For the first approach, searches for the best beam combination can be done using heuristic methods [8, 11], iterative beam angle optimization methods [9, 10, 14, 17, 18] or sparse optimization [19]. For the second approach, beam angle optimization can be done considering derivative-free optimization frameworks [16, 20].

In VMAT, optimization of non-coplanar beam geometries is considered, in most published works, for brain lesions [4, 22–30] and breast/chest wall irradiation [27, 28, 31–33]. Non-coplanar beam optimization for head-and-neck tumours [21, 34–36], lung [25, 29], liver [29] and prostate [27] has also been reported over the past years. The first planning studies using one or more arcs with static couch [23, 31, 33] or planner-defined arc trajectories [4, 21, 32] confirmed the benefits of non-coplanar incidences by OAR sparing. Recently, automated techniques have also been investigated. The simultaneous movement of the gantry and the couch while the beam is being modulated by the continuous movement of the multi-leaf collimator (MLC) grant to the plan optimization process additional degrees of freedom that may result in promising improvements of plan quality. Similarly to the IMRT beam angle optimization problem, the arc trajectory optimization problem can also be divided into two classes of methods: those that decouple arc trajectory optimization from FMO and those that jointly address the two optimization problems. In the first class, geometric and/or dosimetric heuristics [22, 24, 26, 35] are used to define feasible beam orientations. After that, the best delivery trajectory is determined. Beam grouping techniques [22, 26] or graph search techniques, such as those proposed by Dijkstra [24] or the A* algorithms [35] that intend to solve the travelling salesman problem, are used to generate multiple sub-arcs (arcs with static couch or static gantry angles) paths or continuous gantry/couch angle paths, respectively. The VMAT plan is posteriorly optimized along the trajectory in a distinct optimization phase. In the second class of methods, fluence-based methods are used to guide the arc trajectory optimization problem. In some published works, non-coplanar beam angles, obtained from the IMRT fluence-based beam angle optimization problem, are used as anchor points for the path definition. The final arc trajectory is determined by solving the travelling salesman problem [25, 34]. Although promising, these methods do not fully guarantee the optimality of the plan solution over the whole trajectory. Alternatively, techniques combining iteratively sparse solutions of feasible beams with graph search optimization for the trajectory definition [28] or applying Monte Carlo Tree Search algorithms [30] have been proposed. More recently, the anchor point concept was adapted to improve the dosimetric objectives over the whole arc trajectory, by including these optimal incidences in an iterative combinatorial beam angle optimization process that will add new anchor points until the beam path is completely defined [36]. Mixed approaches, that apply methods from both classes during the arc trajectory optimization phases, have also been recently presented [27, 29].

In previous works, we have addressed the static beam angle optimization problem for head-and-neck pathologies [37, 38]. Two beam angle optimization algorithms belonging to the discrete and continuous space search approach optimization classes were compared using a dedicated plan assessment tool [39] also developed by our research group. In the present work, comparison of non-coplanar plans using static beams and arcs for intracranial tumour cases is made. An alternative methodology is used to calculate the non-coplanar trajectories and the BAO search is guided by the outcome ofthe dedicated plan assessment tool. For the intracranial cases, a high level of target coverage and conformity is required for plan approval. Non-coplanar beams or coplanar arcs combined with inverse planning optimization techniques are normally used in the clinical routine. In this study, the potential improvements of the automatic selection of the irradiation directions were investigated for a sample of ten meningioma cases. Algorithms for beam angular optimization and for arc trajectory optimization were applied. A global plan score, based on the dosimetric parameters of the anatomic structures and on the radiation oncologist clinical preferences [39], was used to guide the non-coplanar beam angle optimization problem. For the arc trajectory optimization, a new two-step approach using optimized non-coplanar static beam directions as anchoring points of the arc path was proposed.

## Materials and methods

### Patient data

Ten meningioma cases already treated with stereotactic IMRT were selected for this study. All structures were delineated using two imaging modalities: computed tomography and magnetic resonance images that were conveniently fused. Apart from the planning target volume (PTV), the brainstem, the lens, the retinas, the optical nerves, the chiasm, the pituitary gland and the cochleas were also contoured by the radiation oncologist. The PTV was prescribed with doses of 50.4 Gy, 54.0 Gy, or 59.4 Gy delivered in fractions of 1.8 Gy or with 60.0 Gy delivered in 2.0 Gy fractions. The organs-at-risk (OAR) tolerance doses were established in agreement with the institutional protocol for the intracranial tumours treated with stereotactic IMRT (Table S1 in the Supplementary material).

### Plan generation and optimization

The FMO was performed by Erasmus-iCycle IMRT multicriteria optimization framework [14], that is guided by a wish-list using a constraint-based method (2pεc method) to generate a single Pareto solution in an automatic way [40]. The multicriteria optimization framerwork used is different from the multicriteria approaches based on Pareto surface navigation methods [41]. A pencil-beam dose algorithm with equivalent path length inhomogeneity corrections is used to compute the dose distribution with a beamlet size of 2.5 × 5.0 mm^2^ and with 10 mm of scatter radius. No fluence segmentation is done during or after the optimization phase in Erasmus-iCycle. For VMAT plans, the continuous gantry and MLC motions were approximated by 21 equidistant no sequenced intensity-modulated static beams distributed over the trajectory [42, 43].

The wish-list is composed of a set of clinical constraints and objectives. The constraints must be fulfilled by the multicriterial optimization algorithm and the objectives must be assigned with an optimization priority. For the meningioma cases, the wish-list was composed of six constraints and sixteen prioritized objectives divided into two optimization levels (Table S2 in the Supplementary material). The objective function associated with the PTV was the Logarithmic Tumour Control Probability (LTCP) function, regulated by a cell sensitivity parameter (α). An α value of 0.75 was applied to guarantee good coverage, i.e. that at least 95% of the PTV volume receives the prescription dose (D_p_). The criteria considered for each OAR in the optimization levels were established according to the organ architecture. For the first optimization level, maximum dose objectives for the organs with serial architecture and mean dose objectives for the organs with parallel architecture were considered. For the second level, mean dose and maximum dose objectives were added for the organs with serial and parallel architectures, respectively. The generalized Equivalent Uniform Dose (gEUD) with a value of the tissue-specific parameter that describes the volume effect (*a*) equal to 15 and 6 was also used to minimize the maximum and the mean doses of the lenses and of the cochleas, respectively.

Based on the wish-list template, four types of plans were generated in Erasmus-iCycle:

(i) *CLIN* plans that employed the same beam configurations used by the planners in the clinical routine (4–6 non-coplanar beams manually defined according to the case complexity) to produce IMRT plans;
(ii) *BAO* plans that used five non-coplanar beams optimized by the beam angle optimization algorithm described in Sect. 2.3 to generate IMRT plans;
(iii) VMAT plans that applied 21 equidistant coplanar IMRT static beams to approximate coplanar VMAT plans;
(iv) ATO plans that employed 21 equidistant non-coplanar IMRT static beams to approximate non-coplanar VMAT plans. For these plans, equivalent trajectories were based on five initial non-coplanar anchor points optimized by the proposed arc trajectories optimization process (see Sect. 2.4). To further explore the potential advantage of the arc trajectory optimization approach, an additional plan based on 9 initial anchor points (*ATO9*), was added to the initial plan library set, for a specific patient case.

### Beam angle optimization

The non-coplanar beam angle optimization of IMRT plans was performed using a derivative-free parallel multistart framework approach based on a continuous exploration of the search space to find the best beam ensemble [20]. To prevent possible collisions of the treatment couch and the patient with the gantry, avoidance beam orientations were defined based on clinical experience in the stereotatic treatment of intracranial tumours. In the non-coplanar beam angle optimization algorithm, there is no explicit restriction that prevents the algorithm from reaching these orientations. Instead, avoidance beam orientations are penalized with a very large objective function value and are therefore disregarded.

The adopted beam angle optimization procedure takes advantage of relevant properties of the beam angle optimization search space. One of the main features is the symmetry of the solutions in the beam angle optimization search space due to the simple fact that the order of the beam irradiation directions is irrelevant^16^. This symmetric feature allows a drastic reduction of the space to be searched that can then be divided into several sub-regions allowing a parallel multistart exploration [20]. The optimization problem in each of the defined sub-regions is still a highly non-convex problem with many local minima, so a derivative-free algorithm was chosen to avoid getting trapped in these local minima [44]. The measure used to compare different beam ensembles, and thus to drive the beam angle optimization search, was the SPIDERplan global plan score described in Sect. 2.5. The parallel multistart framework using a derivative-free algorithm guided by this global score is described in more detail in the Supplementary material.

### Arc trajectory optimization

**Fig. 1:**
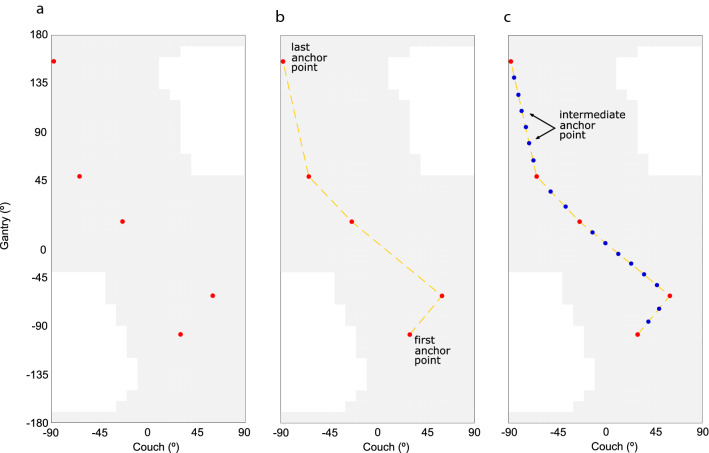
Arc trajectory optimization algorithm phases. **a** 5 non-coplanar beam angle optimized solution that defines the initial anchor points (red dots). **b** linear trajectories (yellow dashed lines) between the anchor points. **c** intermediate anchor points (blue dots) definition (the gantry = -90º and the couch = -90º correspond respectively to a gantry = 270º and a couch = 270º in IEC 61,217 coordinate system)

The non-coplanar arc trajectory optimization was done assuming that the gantry and the couch can rotate simultaneously with different rotation speeds, enabling the definition of highly non-coplanar trajectories. In this work, a two-step approach combining dosimetric considerations and geometric features, was followed. In the first step, the parallel derivative-free multistart framework was used to find feasible non-coplanar beam angles that will be defined as anchor points of the new arc trajectory (red dots in Fig. 1a). In the second step, the anchor points were connected through the definition of linear trajectories (yellow dashed lines in Fig. 1b). New anchor points, placed equidistantly, were added to the trajectory so that the arc was divided into 21 arc sectors (blue dots in Fig. 1c) to mimic a true VMAT technique (in this work, the gantry = -90º and the couch = -90º correspond respectively to a gantry = 270º and a couch = 270º in IEC 61,217 coordinate system).

The gantry and couch movements, defined by the linear paths passing through the anchor points, were outlined taking into account total delivery treatment time. It was established that the gantry trajectory should be always continuous without any inversions in its rotation and never exceeding a 360º arc. For the couch rotation movement, it was also defined that it could be reversed or even halted during the arc delivery. It was also decided that the trajectory must start at the anchor point closest to the search space coordinates (gantry = −80, couch = −90) and, when moving from one anchor point to another, the smallest distance between points must be considered. For anchor points placed inside avoidance regions, a shift in the couch position to the nearest possible beam incidence was done. The final beam trajectory is also tested for possible collisions. For the sections of the trajectory defined inside the avoidance regions, a readjustment of the closest anchor points position is performed.

### Plan assessment and comparison

Plan assessment and comparison was performed with a graphical method named SPIDERplan [39]. This tool uses customised radar plots to graphically display a scoring approach that considers both target coverage and conformity and individual OAR sparing. Depending on their clinical importance, targets and OARs are divided into groups and a score based on the pre-defined planning objectives and relative weights is determined. A global plan score is calculated as a weighted sum of the structures’ individual scores over all groups:

1 $${\text{Global plan score = }}\mathop \sum \limits_{{\text{i }}} {\text{w}}_{{{\text{group}}\left( {\text{i}} \right)}} \mathop \sum \limits_{{\text{j}}} {\text{w}}_{{{\text{struct}}\left( {\text{j}} \right)}} {\text{Score}}_{{{\text{struct}}\left( {\text{j}} \right)}}$$

where w_struct(j)_ and Score_struct(j)_ are the relative weight and the score of structure j, respectively, and w_group(i)_ is the relative weight of group i.

For the PTVs, the coverage and the conformity concepts, normally used by the radiation oncologist to assess the target’s dose distribution for intracranial cases treated with stereotactic irradiation techniques, are included in the score:

2 $${\text{Score}}_{{{\text{PTV}}}} { = }\frac{{1}}{{2}}\left( {\frac{{{\text{D}}_{{\text{TC,PTV}}} }}{{{\text{D}}_{{\text{P,PTV}}} }}{ + }\frac{{{0}{\text{.6}}}}{{{\text{PCI}}}}} \right)$$

where D_TC,PTV_ corresponds to the tolerance criteria for the PTV (in this case the dose in 95% of the PTV that should receive at least the prescribed dose, Table S1 in Supplementary material) and DP,PTV is the planned dose in the PTV. PCI is the Paddick [45] plan conformity index that, for conformal plans, should be above 0.6:

3 $${\text{PCI = }}\frac{{{\text{V}}_{{{\text{PTV,100\% }}}}^{{2}} }}{{{\text{V}}_{{{\text{PTV}}}} {\text{ V}}_{{{\text{External,100\% }}}} }}$$

where V_PTV,100%_ is the volume of the PTV covered by the isodose prescription, V_PTV_ is the volume of the PTV and V_External,100%_ is the volume of the body covered by the isodose prescription.

For the OARs, the score was set as:

4 $${\text{Score}}_{{{\text{OAR}}}} { = }\frac{{{\text{D}}_{{\text{P,OAR}}} }}{{{\text{D}}_{{\text{TC,OAR}}} }}$$

where D_P,OAR_ is the OAR planned dose and D_TC,OAR_ is the tolerance dose for each OAR. For each objective, a value of one is expected if the dose for that structure is equal to the respective tolerance value. When a better organ sparing or target coverage is obtained, a score less than one will be obtained.

For this study, all delineated structures were grouped according to their location and clinical importance. Therefore, the PTV was assigned to the PTV group with a relative weight of 40%, the brainstem to the Critical group with a relative weight of 50%, the chiasm, the optical nerves, the retinas and the lens to the Optics group with a relative weight of 7% and the cochlea and pituitary gland to Other group with a relative weight of 3%. Within each group, the same weight was attributed to all its structures (Table S2 in the Supplementary material). A partial group score based on the dose sparing of the structures that belong to that group was also calculated.

SPIDERplan analysis was complemented by the gradient index (GI) proposed by Paddick and Lippitz [46]. The GI is a quality index used in the clinical routine to assess the quality of stereotactic brain cases that measures the steepness of the dose gradient outside the PTV providing information about the amount of irradiated healthy tissue. The GI is given by:

5 $${\text{GI = }}\frac{{{\text{V}}_{{{\text{External,50\% }}}} }}{{{\text{V}}_{{{\text{External,100\% }}}} }}$$

where V_External,50%_ corresponds to the volume of healthy tissue covered by the isodose surface corresponding to half of the prescription dose. The lower the GI value, the higher the dose gradient and the sparing of healthy tissue near the PTV.

### Statistical analysis

Statistical comparisons of the global plan and the group scores were performed with IBM SPSS software, version 25. Statistically significant differences between the plan sets were assessed using a randomized block design ANOVA test and, if applicable, a post-hoc multiple comparison test using the Tukey method. For all statistical tests a level of significance of 5% was considered.

## Results

**Fig. 2:**
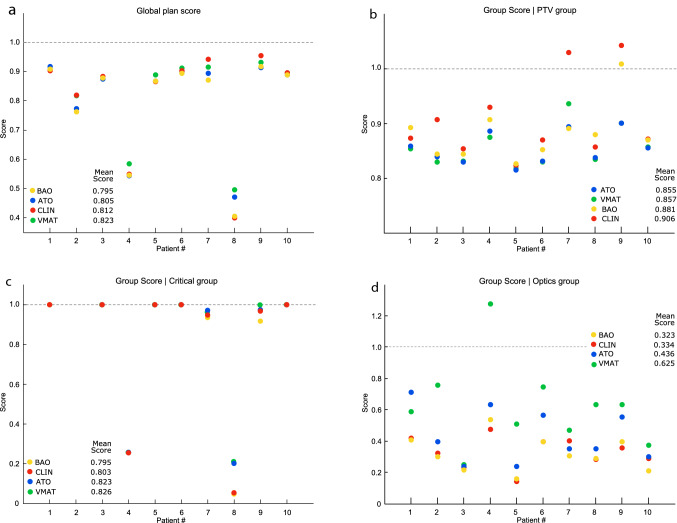
SPIDERplan analysis of the *CLIN*, *BAO*, *VMAT* and *ATO* plans for the 10 meningioma cases. a1) SPIDERplan global plan score. **b** SPIDERplan group scores for the PTV group. **c** SPIDERplan group scores for the Critical group. **d** SPIDERplan group scores for the Optics group

The SPIDERplan global plan score values of *CLIN*, *BAO*, *VMAT* and *ATO* plans for all meningioma cases are shown in Fig. 2a. All plans presented global plan scores well below unity as a result of the high-quality level of the obtained dose distributions (see Fig. S1 and S2 in the Supplementary material where an example is shown). The mean global plan scores over all patients ranged between 0.795 (for *BAO*) and 0.823 (for *VMAT*).

The statistical analysis applied to the global plan scores of the plan sets is summarized in Table S3 in the Supplementary material. Statistically significant differences between the global plan scores of the plan sets were found with the randomized block design ANOVA test (p-value = 0). Pairs of plan sets which do not statistically differ from each other were identified by the post-hoc multiple comparison test applied with the Tukey method. Two subsets, grouping the plan sets which did not present statistically significant differences were built. The first subset included the *BAO*, the *ATO* and the *CLIN* sets, meaning that the quality of these plans is statistically equivalent. The second subset grouped the *VMAT, the ATO and* the *CLIN* sets. Statistically significant differences in plan quality were only found between the *BAO* plans and the *VMAT* plans.

The results of the group scores are shown in Fig. 2b for the PTV group, in Fig. 2c for the Critical group and in Fig. 2d for the Optics group. On average, all sets presented mean group scores below one, corroborating the assessment results of the global plan score. For the PTV group, the best coverage and conformity indexes were achieved by the arc-based plans (*ATO* and *VMAT*), while for the Critical group (with the highest weight group) and for the Optics group the best scores were achieved for the static beams-based plans (*BAO* and *CLIN*).

**Fig. 3:**
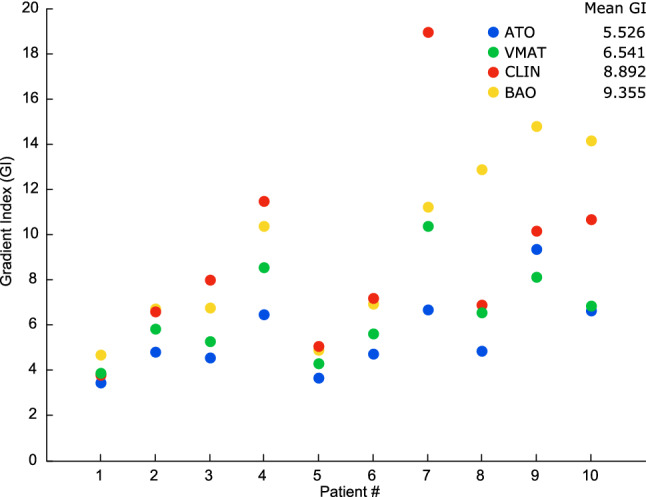
Gradient index computed for the 10 patient cases

The evaluation of the steepness of the dose falloff outside the PTV was performed through the determination of GI (Fig. 3). The arc-based plans *(ATO* and *VMAT)* presented the lower mean values of GI, while the plans optimized with static beams (*CLIN* and *BAO*) presented the highest mean values of GI. For similar PTV coverage and conformity, the volume of healthy tissues receiving a dose between half of the prescription and the prescription dose is, on average, two times larger for static beam plans.

**Fig. 4:**
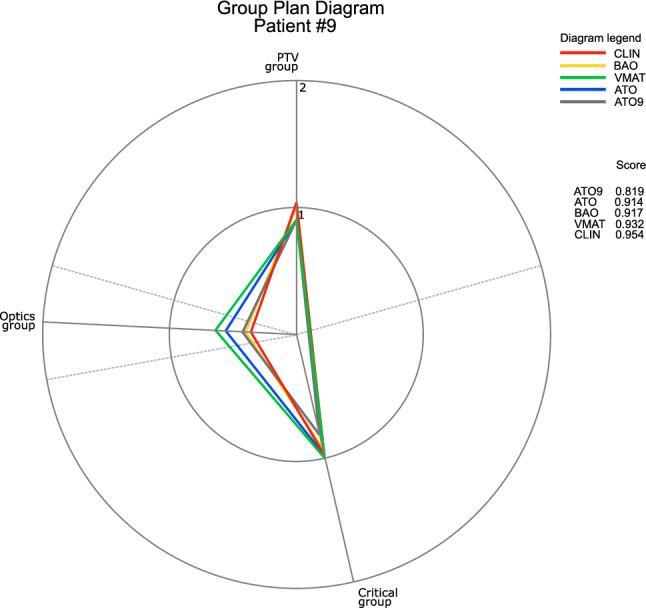
SPIDERplan of patient case number 9

In radiation therapy, the inherent patient-specificity usually requires a careful evaluation of the available treatment options. Among the 10 cases of our sample, one (patient #9), presented significant differences in SPIDERplan scores (global plan score and group scores) and in the gradient index values. For this patient, the PTV, located next to the chiasm, the left optic nerve and the brainstem, was prescribed with a dose of 60 Gy. In addition to the initial set of plans, an arc trajectory optimized plan based on 9 anchor points (*ATO9*) was also calculated for this specifc patient. The evaluation of the quality of the plans calculated for patient #9 is presented in Fig. 4. For this patient, the best plans were achieved by techniques with direction/trajectory optimization (*ATO9*, *ATO* and *BAO*) and the worst by the *CLIN* plan. *ATO*-based plans achieved a high level of coverage and conformity of the PTV, while the static beam-based plans presented better performance for the OARs groups. Good results were also achieved by *ATO9.* For the Critical group, *ATO9* presented the best group score and improved the sparing of the Optics group compared to *ATO5*. In fact for this patient, the increase from 5 to 9 anchor points allowed an improvement of 10% in the global score plan. These results highlight not only the potential benefits that may arise from the optimization of direction/trajectory of the beams but also the need of investigating in more detail the influence of the number of anchor points on the quality of the dose distribution.

## Discussion

In this work, algorithms for non-coplanar beam angular optimization and non-coplanar arc trajectory optimization were applied to 10 meningioma cases. Beam angular optimization was based on a multistart derivative-free optimization framework and guided by SPIDERplan global plan score. These non-coplanar static beam angle optimized incidences were used for non-coplanar VMAT plans generation, defining the anchor points of linear trajectories connecting consecutive points.

The fluence-based beam angle optimization methods are usually guided by the objective function values of the FMO problem, guaranteeing that reliable and high-quality plans are found. However, it cannot be assumed that a plan calculated by these optimization procedures will be selected or even approved by the radiation oncologist. The integration of SPIDERplan global plan score in the beam angle optimization problem, as a measure of the quality of the beam angle set, intended to generate a plan solution that is optimal from the inverse planning optimization point of view. This solution should also be able to fulfil the clinical aims defined by the radiation oncologist. The referred methodology was firstly applied by Rocha et al*.* [20] to nasopharynx cancer cases, where the quality of the plans generated with and without the global plan score guidance was compared. The plans optimized with the global plan score presented a higher sparing of the OARs for the same PTV coverage than the ones optimized with the common objective function. In the present study, the global plan score was used to guide the optimization of the irradiation directions of the *BAO* and the *ATO* plans for intracranial tumours. As in Rocha et al*.* [20], the application of the global plan score to the beam angle optimization problem produced plans with excellent levels of PTV coverage and conformity and high sparing of the OARs.

The arc trajectory optimization problem is often mathematically described as a problem even more complex than the beam angle optimization problem [25, 29]. The two-step method presented in this work intends to take advantage of the experience and the knowledge acquired with the beam angle optimization problem [16, 20, 36–38] contributing to the scientific debate of the arc trajectory optimization problem. The first step of the proposed arc trajectory method consisted in identifying the anchor points of the beam trajectory. The number and location of these points should guarantee a proper sampling of the space search and a smooth beam arc trajectory. A very high number of anchor points could result in complex or irregular trajectories when a connection method is applied while a very low number of anchor points may not be sufficient to define a trajectory likely to generate good quality plans. These initial configuration issues were handled by defining a fixed number of beams to be used in the optimization and by selecting a derivative-free optimization algorithm that considers a continuous search space for optimization. According to our recent work [37], this algorithm presents a good performance on non-coplanar optimization geometries and a good beam coverage of the space search.

The number of beams defined for the non-coplanar beam angle optimization was based on clinical experience. For the ten meningioma cases considered for this study, the planners need, on average, 5 IMRT beams to achieve a satisfactory plan. In the second step of the arc trajectory algorithm, where the shape of the arc trajectory was defined, the anchor points were connected through linear paths. The rationale behind this choice was a literal interpretation of the term ‘anchor point’ and an option for simplifying the optimization algorithm. The irradiation directions that composed the linear trajectory between the anchor points were not fluence-based optimized. The linear trajectory between the optimal 5-beam configuration is not guaranteed to be better than a linear trajectory of another set of five initial anchor points. That would be also true for any other type of trajectory between the initial anchor points. But starting from optimized directions will possibly lead to improved trajectories. However, this drawback was overcome by using an efficient inverse planning optimizer as the Erasmus-iCycle multicriteria engine. The connection order of the linear paths was established by following the pre-condition that the gantry movement was continuous, inversions were not allowed and it was not possible to exceed a 360º arc. It was assumed that the gantry and the couch speeds may be different, and that the inertia associated with the change of velocity or direction of the couch is much lower than the inertia of the gantry. The implementation of these trajectory configuration options was only possible due to the regular and well disperse beam angle distribution of the anchor points in the search space that resulted from the non-coplanar beam angle optimization.

A VMAT optimization module was not available in Erasmus-iCycle at the time the arc trajectory optimization was performed. The continuous motion of the gantry and the MLC in the VMAT delivery were approximated by using 21 equidistant static beams distributed over the trajectory as was demonstrated by Bortfeld [42]. This approach implies that the beam modulation that occurred during each arc sector of ~ 17º is replaced by an intensity-modulated field placed in the centre of that arc sub-sector. The conversion of these calculated fluence maps into deliverable MLC segments over all the arc trajectory could be done using arc sequencing methods. They will inevitably lead to dose degradation due to the distribution of the sequenced apertures over the arc trajectory sectors defined by the anchor points [43]. An alternatively solution, widely implemented,is based on algorithms that combine arc sequencing methods with direct aperture optimization methods, such those proposed by Wild et al*.* [34] and Bzdusek et al*.* [47]. The use of direct aperture optimization algorithms based on gradient methods after the arc sequencing methods will refine the leaf apertures optimization and could improve the plan quality [43]. In the future, improvements to this arc trajectory optimization method should include endorsing the process of fully fluence-based methods like the one proposed by Rocha et al. in a preliminary study [36] and by using a VMAT optimization algorithm to calculate an improved dose distribution.

The plans optimized with non-coplanar irradiation directions, *CLIN, BAO and ATO*, presented a lower global plan score, which corresponds to a slight higher plan quality, than the plans based on coplanar geometries (*VMAT*). The advantages of non-coplanar beams geometries over coplanar ones for brain cases were previously reported [3, 8, 9]. However, consensus about the best radiation therapy technique to treat these tumour lesions was not reached. Fogliata et al*.* [48] reported equivalent quality between non-coplanar IMRT plans, coplanar VMAT plans and helical Tomotherapy plans. Conversely, Panet-Raymond et al*.* [49] achieved equivalent PTV coverage with non-coplanar IMRT and coplanar VMAT plans, but higher OAR sparing with the former set of plans.

For non-coplanar geometries, all plans achieved high-quality (average SPIDERplan global scores were well below one). Although the best performance was obtained by *BAO,* no statistically significant score differences were found, on average, between *BAO*, *ATO* and *CLIN* plans. The *BAO* plans were most effective in sparing the OARs, while the *ATO* plans enabled higher PTV coverage and conformity. Furthermore, a steeper dose gradient outside the PTV was also possible with the *ATO* plans, due to the higher number of irradiation directions available with this technique. Previous studies with non-coplanar arc trajectory optimization algorithms applied to intracranial tumours have been published. Wild et al*.* [34] applied, for three nasopharynx tumour cases, a genetic algorithm to determine the static anchor points and defined the beam trajectory by calculating the shortest delivery time between these points. Papp et al*.* [25] applied an iterative beam angle optimization method to define the anchor points and solved the travelling salesman problem to define the remaining trajectory of the arc for a lung and a brain tumour. Langhans et al. [29] used, in a lung, a liver and a brain cases, an iterative method based on a 4π solution to find the feasible anchor points and defined the arc trajectory based on geometrical scoring evaluation of the available beam directions. All works reported improved plan quality when non-coplanar VMAT plans were compared with non-coplanar IMRT plans (equivalent to *CLIN* plans). Although these conclusions are in line with the results of this study, our *ATO* plans have not brought any additional improvements to the quality of the dose distribution, when compared with *BAO* plans. This finding may be related with the high performance of the non-coplanar beam angle optimization algorithm and the multicriterial IMRT optimization engine, that are able to generate high-quality plan solutions with a low number of static beam directions (5 non-coplanar beams). Furthermore, the number of anchor points selected to build the beam trajectory may not be optimal. As was shown in the analysis of patient #9, for some more complex situations, a higher number of anchor points can be advantageous. This result is also in agreement with the findings of Wild et al*.* [34]. The authors showed that, until a given unspecified limit, a larger number of initial static trajectory positions may result in plan dose distribution quality improvements. For patient #9, it was decided to run the arc trajectory optimization based on 9 anchor points rather than considering a lower number of initial anchor points. This decision was based on the results of our previous work for coplanar and non-coplanar static beam angular optimization for nasopharynx cases [37], that showed no significant statistical differences between 5 and 7 beams plans. However, significant improvements in the dose distribution quality were found when increasing the number of beams from 5 to 9. The determination of the ideal number of anchor points for specific case applications is out of the scope of the present work, presenting an interesting challenge to be tackled in future work.

Higher gradient index values were obtained for *CLIN* and *BAO* plans than for *ATO* and *VMAT* plans. Furthermore, only weak or very weak associations were found between the GI and the prescription dose or the global plan score. This confirms that, in general, the dose falloff outside the target is mainly determined by the irradiation technique.

The high quality of *CLIN* plans, and the fact that no statistically significant differences between the score of the *BAO* and the *ATO* plans were obtained, must be highlighted. In the clinical routine, the plans for patients with intracranial tumours are usually manually done with 4–6 non-coplanar beams in a very time-consuming process, attempting to spare as much as possible the OARs and to fulfil the PTV coverage requirements. This planning strategy is clearly shown by the group score results that present similar behaviour to the *BAO* plans, i.e. better score in the OARs groups than in the PTV group.

The inclusion of non-coplanar beam angle optimization and arc trajectory optimization into inverse treatment planning inevitably leads to increased optimization times. Even so, the potential treatment plan quality improvements and the integration of plan optimization engines with minimal intervention from the user and with the guarantee of consistent generation of high-quality plans (such as the *BAO* and the *ATO* plans) should motivate a strong commitment towards introducing automated planning tools in the clinical practice.

## Conclusions

In this work, a beam geometry with five non-coplanar incidences was chosen to run the beam angle and the arc trajectory optimization problem for the intracranial pathology using ten meningioma cases. BAO plans presented, on average, a lower global plan score than ATO and CLIN plans, but without statistically significant differences. The *ATO* plans assured a more efficient coverage and conformity of the PTV, while a higher sparing of the OARs was achieved by the *BAO* plans. This global analysis does not dismiss an individual patient analysis, where strong benefits may be obtained with 4π directions optimization in specific patients.

## Supplementary Information

Below is the link to the electronic supplementary material.

Supplementary file1 (DOC 4112 kb)
