# Supplementary material for: Comparison of non-coplanar optimization of static beams and arc trajectories for intensity-modulated treatments of meningioma cases
Source: Phys Eng Sci Med. 2021 Oct 7;44(4):1273–83. doi: 10.1007/s13246-021-01061-8 (PMC8668856; doi:10.1007/s13246-021-01061-8)
Supplement: Supplementary file 1 — Supplementary file1 (DOC 4112 kb) [file 13246_2021_1061_MOESM1_ESM.doc]

**Comparison of non-coplanar optimization of static beams and arc trajectories for intensity-modulated treatments of meningioma cases**

**Physical and Engineering Sciences in Medicine**

Tiago Ventura ^1,2,3^, Humberto Rocha^3,4^, Brigida da Costa Ferreira^3,5,6^, Joana Dias^3,4^, Maria do Carmo Lopes^2,3,6^

^1^ Physics Department of University of Aveiro, Aveiro, Portugal

^2^ Medical Physics Department of the Portuguese Oncology Institute of Coimbra Francisco Gentil, EPE, Coimbra, Portugal

^3^ Institute for Systems Engineering and Computers at Coimbra, Coimbra, Portugal

^4^ Economy Faculty of University of Coimbra and Centre for Business and Economics Research, Coimbra, Portugal

^5^ Instituto de Biofísica e Engenharia Biomédica, Faculdade de Ciências da Universidade de Lisboa, Portugal

^6^ I3N Physics Department of University of Aveiro, Aveiro, Portugal

**Corresponding author and address:**

Tiago Ventura

tiagoventura@ipocoimbra.min-saude.pt

**Supplementary material**

Table S1 – Groups, structures and weights considered for SPIDERplan processing.

| **Groups** | |  | **Structures** | |  |
| --- | --- | --- | --- | --- | --- |
| *Name* | *Group Weight* |  | *Name* | *Structure*  *Weight* | *Tolerance criteria* |
|  |  |  |  |  |  |
| PTV | 40% |  | PTV | 100% | V_100%_ ≥ 0.95 |
|  |  |  |  |  | PCI > 0.6 |
|  |  |  |  |  |  |
| Critical | 50% |  | Brainstem | 100% | D_max_ ≤ 54Gy |
|  |  |  |  |  |  |
| Optics | 7% |  | Chiasm | 14.3% | D_max_ ≤ 55Gy |
|  |  |  | Left optic nerve |  | D_max_ ≤ 55Gy |
|  |  |  | Right optic nerve |  | D_max_ ≤ 55Gy |
|  |  |  | Left retina |  | D_max_ ≤ 45Gy |
|  |  |  | Right retina |  | D_max_ ≤ 45Gy |
|  |  |  | Left lens |  | D_max_ ≤ 12Gy |
|  |  |  | Right lens |  | D_max_ ≤ 12Gy |
|  |  |  |  |  |  |
| Other | 3% |  | Right cochlea | 33.3% | D_mean_ ≤ 45Gy |
|  |  |  | Left cochlea |  | D_mean_ ≤ 45Gy |
|  |  |  | Pituitary gland |  | D_max_ ≤ 60Gy |
|  |  |  |  |  |  |
| D_max_ - maximum dose, D_mean_ - mean dose, V_100%_ - volume of the prescription dose surface, PCI - plan complexity index | | | | | |

Table S2 – General wish-list defined for meningioma cases.

| **Level** |  | **Priority** |  | **Structure** |  | **Type** |  | **Goal** |  | **Sufficient** |  | **Parameters** |
| --- | --- | --- | --- | --- | --- | --- | --- | --- | --- | --- | --- | --- |
|  |  |  |  |  |  |  |  |  |  |  |  |  |
| **Constraints** |  |  |  |  |  |  |  |  |  |  |  |  |
|  |  |  |  | PTV |  | maximum |  | D_p,107%_ |  |  |  |  |
|  |  |  |  | Brainstem |  | maximum |  | 54 Gy |  |  |  |  |
|  |  |  |  | Retinas |  | maximum |  | 45 Gy |  |  |  |  |
|  |  |  |  | Optical nerves |  | maximum |  | 55 |  |  |  |  |
|  |  |  |  | Chiasm |  | maximum |  | 55 |  |  |  |  |
|  |  |  |  | Unspecified Tissue |  | maximum |  | D_p_ |  |  |  |  |
|  |  |  |  |  |  |  |  |  |  |  |  |  |
| **Objectives** |  |  |  |  |  |  |  |  |  |  |  |  |
| 1 |  | 1 |  | PTV |  | LTCP |  | 1 |  | 0.5 |  | D_p_/0.95 Gy, α=0.75 |
|  |  | 2 |  | Brainstem |  | maximum |  | f_10_DVH |  |  |  |  |
|  |  | 3 |  | Chiasm |  | maximum |  | f_10_DVH |  |  |  |  |
|  |  | 4 |  | Optical nerves |  | maximum |  | f_10_DVH |  |  |  |  |
|  |  | 5 |  | Retinas |  | maximum |  | f_10_DVH |  |  |  |  |
|  |  | 6 |  | Lens |  | gEUD |  | f_10_DVH |  |  |  | a=15 |
|  |  | 7 |  | Cochleas |  | maximum |  | f_10_DVH |  |  |  |  |
|  |  | 8 |  | Pituitary gland |  | maximum |  | f_10_DVH |  |  |  |  |
|  |  | 9 |  | Ring1 PTV_Dp_ |  | maximum |  | D_p,85%_ |  |  |  |  |
|  |  | 10 |  | Ring2 PTV_Dp_ |  | maximum |  | D_p,70%_ |  |  |  |  |
|  |  | 11 |  | Ring3 PTV_Dp_ |  | maximum |  | D_p,55%_ |  |  |  |  |
|  |  |  |  |  |  |  |  |  |  |  |  |  |
| 2 |  | 12 |  | Brainstem |  | mean |  | f_10_DVH |  |  |  |  |
|  |  | 13 |  | Chiasm |  | mean |  | f_10_DVH |  |  |  |  |
|  |  | 14 |  | Optical nerves |  | mean |  | f_10_DVH |  |  |  |  |
|  |  | 15 |  | Retinas |  | mean |  | f_10_DVH |  |  |  |  |
|  |  | 16 |  | Lens |  | gEUD |  | f_10_DVH |  |  |  | a=6 |
|  |  | 17 |  | Cochleas |  | mean |  | f_10_DVH |  |  |  |  |
|  |  | 18 |  | Pituitary gland |  | mean |  | f_10_DVH |  |  |  |  |
|  |  | 19 |  | External ring |  | maximum |  | 40%×D_p_ |  |  |  |  |
|  |  | 20 |  | Unspecified Tissue |  | mean |  | 5 Gy |  |  |  |  |
|  |  |  |  |  |  |  |  |  |  |  |  |  |
| Ring1 PTV - ring of 5 mm of thickness at 10 mm distance from PTV, Ring2 PTV - ring of 10 mm of thickness at 15 mm distance from PTV, Ring3 PTV - ring of 20 mm of thickness at 25 mm distance from PTV, External ring - ring of 20 mm thickness next to patient outer contour, LTCP - logarithmic tumour control probability, f_10_DVH - dose value calculated with the feasibility curve of 10% from the feasibility module of PlanIQ v2.2 from Sun Nuclear, gEUD - generalized equivalent uniform dose. | | | | | | | | | | | | |

**Algorithm of Beam Angle Optimization**

*Initialization*

- Set
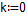
;
- Choose initial points, one for each sub-region,
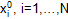
;
- Compute
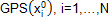
 in parallel;
- Set
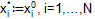
and
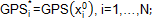

- Set
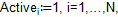
 i.e. all sub-regions initially have active local searches;
- Choose
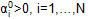
and
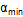
;

*Iteration*

1. Use a derivative-free algorithm to locally explore the sub-regions with active local searches;
2. For sub-regions
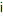
 with active local search do

If
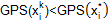
 then

If
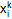
 is in sub-region
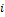
 then


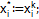


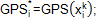


Else


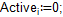


Determine sub-region
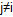
 where
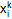
 is;

If
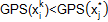
 then


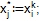


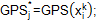


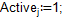


Else


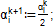


If
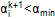
 then


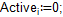


1. If there exist active sub-regions go to first step and set
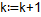
.

(GPS – Global Plan Score;
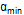
– threshold value)

Table S3 – Statistical analysis using ANOVA with randomized block design and the pos-hoc multiple comparison test with the Tukey method for the global plan score, the PTV group score, the Critical group score and for the Optics group score

| **ANOVA | Randomized block design** | | | | | | | | | | | | | |
| --- | --- | --- | --- | --- | --- | --- | --- | --- | --- | --- | --- | --- | --- |
|  | **Global plan score** | |  | **PTV group score** | | |  | **Critical group score** | |  | **Optics group score** | | |
| p-value | 0.000 | |  | 0.001 | | |  | 0.125 | |  | 0.000 | | |
|  |  | |  |  | | |  |  | |  |  | | |
| **Post-hoc multiple comparison test | Tukey method | Homogeneous subsets** | | | | | | | | | | | | | |
| **Global Plan score** | | |  | **PTV group score** | | |  | **Critical group score** | |  | **Optics group score** | | |
|  | **1** | **2** |  |  | **1** | **2** |  |  | **1** |  |  | **1** | **2** |
| **BAO** | 0.795 |  |  | **ATO** | 0.855 |  |  | **BAO** | 0.795 |  | **BAO** | 0.323 |  |
| **ATO** | 0.805 | 0.805 |  | **VMAT** | 0.857 |  |  | **CLIN** | 0.803 |  | **CLIN** | 0.334 |  |
| **CLIN** | 0.812 | 0.812 |  | **BAO** | 0.882 | 0.882 |  | **ATO** | 0.823 |  | **ATO** | 0.436 |  |
| **VMAT** |  | 0.823 |  | **CLIN** |  | 0.906 |  | **VMAT** | 0.826 |  | **VMAT** |  | 0.625 |

Fig. S1 - Dose distribution for a typical patient generated in CLIN, VMAT, BAO and ATO plans.


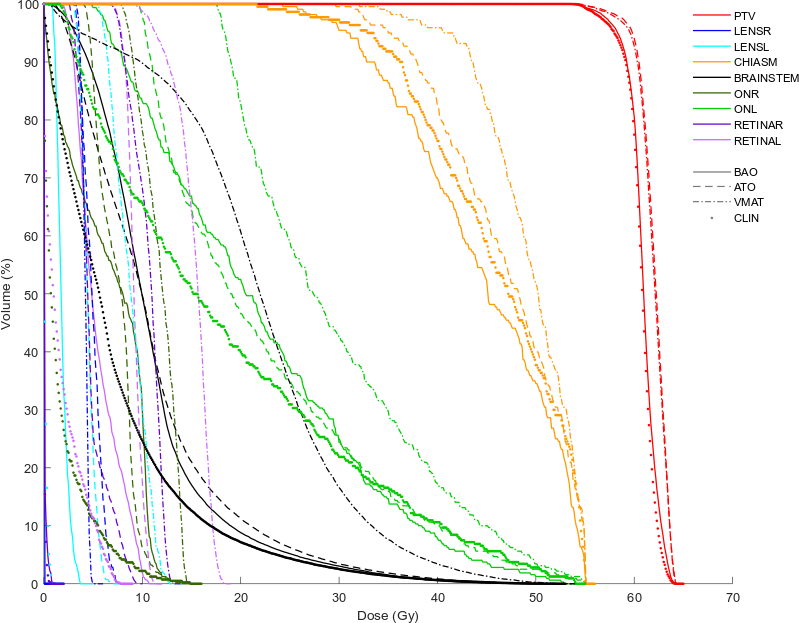


Fig. S2 – Dose volume histogram for a typical patient
